# Arthrodesis of proximal inter-phalangeal joint for hammertoe: intramedullary device options

**DOI:** 10.1007/s10195-015-0360-0

**Published:** 2015-06-27

**Authors:** Matteo Guelfi, Andrea Pantalone, Janos Cambiaso Daniel, Daniele Vanni, Marco G. B. Guelfi, Vincenzo Salini

**Affiliations:** Orthopaedic and Traumatology Division, G. d’Annunzio University, Via dei Vestini 35, 66013 Chieti, Italy; Department of Plastic, Aesthetic and Reconstructive Surgery, Medical University of Graz, Augenbruggerplatz 29, 8036 Graz, Austria; Orthopaedic Division, Clinica Montallegro, Via M.Te Zovetto 27, 16145 Genoa, Italy; Via Caprera 7/3, 16146 Genoa, Italy

**Keywords:** Hammertoe, Arthrodesis, PIP joint, Fusion, Intramedullary devices, Review, K-wire

## Abstract

**Background:**

Proximal inter-phalangeal (PIP) joint arthrodesis today represents the standard treatment for structured hammertoes; however, recently, a lot of new intramedullary devices for the fixation of this arthrodesis have been introduced. The purpose of this work is to look at the currently available devices and to perform a review of the present literature.

**Materials and methods:**

A literature search of PubMed/Medline and Google Scholar databases, considering works published up until September 2014 and using the keywords: hammertoe, arthrodesis, PIP joint, fusion, intramedullary devices, and K-wire, was performed. The published papers were included in the present study only if they met the following inclusion criteria: English articles, arthrodesis of PIP joints for hammertoes with new generation intramedullary devices, series with *n* > 10. Studies using absorbable pins or screws that are considered as another kind of fixation that involved more than one articulation, as well as comments, letters to the editor, or newsletters were excluded.

**Results:**

Nine publications were included. Of the patients’ reports, 93–100 % were good or excellent concerning satisfaction. Radiological arthrodesis was achieved in 60.5–100 % of cases. Three of the publications compared the new devices with the K-wire. Of these three articles, two employed the traditional technique and one the buried technique. The AOFAS score, evaluated in three publications, showed a delta of 19, 45 and 58 points. Major complications, which required a secondary surgical revision, were between 0 and 8.6 %. The complications of the K-wire and the new devices were similar; also the reoperation rate was close to equal (maximal difference 2 %). On the other hand, these kinds of devices definitely have a higher price, compared to the K-wire.

**Conclusion:**

The use of these new devices provides good results; however, their high price is currently a problem. For this reason, cost-benefit studies seem to be necessary to justify their use as standard treatment.

**Level of evidence:**

Level III systematic review.

## Introduction

Nowadays, the treatment of the hammertoe is still disputable; indeed, a lot of procedures, both on the soft tissues and the bone structures are purposed and considered efficient. In the rigid and structured deformities not suited for manual correction, arthrodesis of the proximal inter-phalangeal (PIP) joint represents the standard treatment [1]. This procedure is performed by removing the articular surfaces of the proximal and intermediate phalanges. Many systems such as cannulated screws or absorbable pins have been designed for the fixation of the arthrodesis, yet still the K-wire is the traditional method, and most utilized [2–6]. However, recently, new intramedullary devices have been used persistently, trying to solve problems such as infections [3, 7], traumatic breaks [8, 9] and malalignments [10] tied to the K-wire.

As of today (September 2014), after accurate research, 16 different devices are available on the United States (US) and European (EU) markets. These were divided into four categories according to technical features and material composition (Table 1).

**Table 1:** Intramedullary devices available on US and EU markets (up to September 2014)

Category	Name	Company	Material	No. of sizes available	Plantar angle
Shape memory	Smart Toe^®^ II	Stryker^®^	Memometal Nitinol	6 + 2 × DIP	0°–10°
	Hammerlock^®^	BME^®^	Memometal Nitinol	4 + 1 × DIP	0°–10°
One-piece solid or cannulated	ProToe VO^®^	Wright^®^	Stainless steel	5	0°–10°
	Arrow-lok™	Arrowhead Medical^®^	Stainless steel	8	0°–10°
	Ipp On^®^	Integra^®^	Stainless steel	2	0°
	Proxifuse™	Cartiva^®^	Nitinol and PEEK	1	0°
	Phalinx^®^	Wright™	Titanium	4	0°–10°^a^
	Digifuse™	Metasurg^®^	Titanium	2 + 1 × DIP	0°–10°
	Two Step Imp. Syst.	Trilliant Surgical LTD^®^	Titanium	3	0°
	DuaFit^®^	In 2 Bones	PEEK	4	0°–10°–17°^a^
	Toegrip^®^	Synchro Medical^®^	PEEK	5	0°–10°–20°
	HammerFix^®^	Extremity Medical™	PEEK	3	0°
Bone allograft	TenFuse^®^	Solana Surgical^®^	Bone allograft	2	0°–10°
Two-piece	Stayfuse™	Tornier^®^	Titanium	3 Prox/6mid	0°
	Nextra^®^	Nextremity Solutions^®^	Titanium	2 Prox/3 mid	10°
	Hat-Trick^®^	Smith and Nephew^®^	PEEK	4 Prox/2 mid	0°–10°

^a^10° and 17° angolated are solid, not cannulated

- Shape memory devices: these are composed of a memory metal (Memometal NiTinol), which is activated by body temperature, modifying its shape once implanted. Specifically, these become shorter and enlarge themselves to bestow more stability to the system.
- Bone allograft devices: since these devices are grafts, they have bone inductive and conductive properties, which improve their integration significantly.
- One-piece solid or cannulated devices: thanks to the form of their extremities, these can be anchored to the cortical of the proximal and middle phalanges. The cannulated type also permits the use of the K-wire as a guide. With these devices the proximal part is threaded and screwed onto the proximal phalange, while the distal part is anchored to the middle phalange. They are available in steel, titanium or polyetheretherketone (PEEK).
- Two-piece devices: a female and a male part make up these devices. Once positioned, one on the proximal phalanges, and the other one on the middle, these are fixed together.

The purpose of this work is to look at the currently available devices and to review, from the literature, the results of these for PIP fusion.

## Materials and methods

A literature search of PubMed/Medline and Google Scholar databases, considering works published up until September 2014, and using the keywords: hammertoe, arthrodesis, PIP joint, fusion, intramedullary devices, and K-wire was performed. The published papers were included in the present study only if they met the following inclusion criteria: English articles, arthrodesis of PIP joints for hammertoes with new generation intramedullary devices, series with *n* > 10. Studies using absorbable pins or screws that are considered as another kind of fixation that involved more than one articulation, as well as comment, letter to editor or newsletters were excluded.

The search strategy identified over 455 articles. A total of 43 publications describing specifically the arthrodesis of the PIP joint for hammertoe could be identified.

**Fig. 1:**
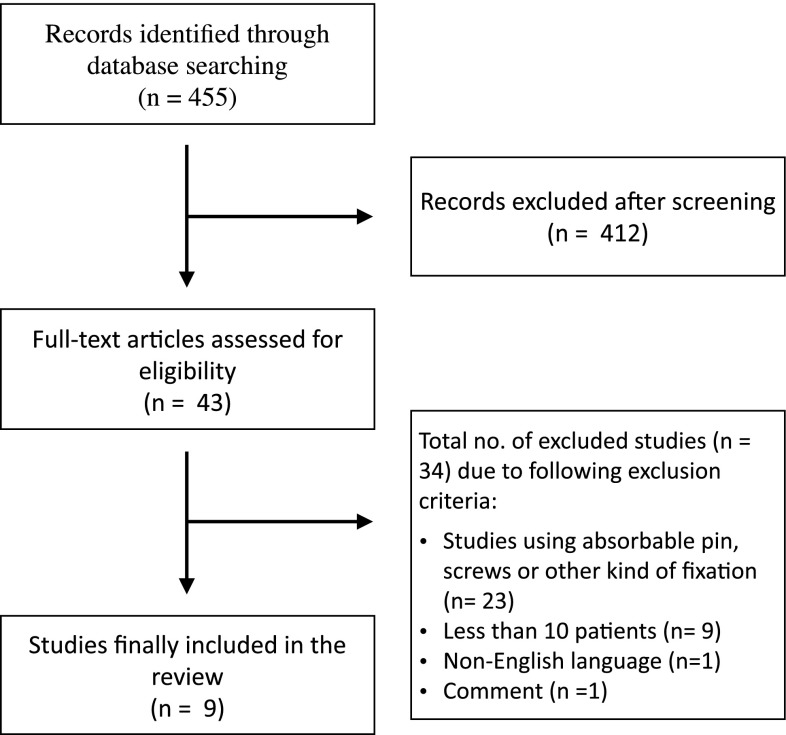
Flowchart of eligible study evaluation

Thirty-four articles were excluded due to exclusion criteria, these being: studies using absorbable pins, screws or other kinds of fixation (*n* = 23), fewer than ten patients (*n* = 9), non-English language (*n* = 1), comment (*n* = 1) (Fig. 1).

Nine articles, finally, met the inclusion criteria and were compatible with our review (Table 2) [5, 10–17]. In these papers, patient satisfaction, achievement of arthrodesis, AOFAS score and the rate of complications were taken into consideration.

**Table 2:** Results of the reviewed articles

References	Level of evidence	Device	Toes/no. patients	Patients’ satisfaction (%)	Acceptable alignment (%)	Radiological arthrodesis (%)	AOFAS variation (preoperative/postoperative)	K-wire revision surgery (%)	Device revision surgery (%)
Angirasa et al. *J Foot Ankle Surg* [11]	IV, retrospective case series	Smart Toe^®^	13/13	98.3	NA	100	NA	0	0
Catena et al. *Foot Ank Int* [12]	IV, case series	Smart Toe^®^	42/24	100	100	81	19 (52/71)	NA	0
Coillard et al. *Foot Ank Int* [13]	IV, prospective case series	Ipp-On^®^	156/117	98	95.9	83.8	45 (40.4/84.3)	NA	0.6
Ellington et al. *Foot Ank Int* [14]	IV, retrospective case series	Stayfuse^®^	38/27	NA	81.6	60.5	NA	NA	7.9
Fazal et al. *Foot Ank Int* [15]	IV, retrospective case series	StayFuse^®^	150/140	95	NA	73	58.7 (22.9/81.6)	NA	3.3
Roukis et al. *Foot Ankle Spec* [10]	IV, retrospective case series	Smart Toe^®^	30/10	NA	NA	93	NA	0	0
Sandhu et al. *Foot Ankle Spec* [16]	IV, case series	Smart Toe^®^	65/35	NA	NA	93.8	NA	NA	0
Scholl et al. *J Foot Ankle Surg* [17]	IV, retrospective case series	Smart Toe^®^	58/NA	NA	87.9	68.9	NA	10.7	8.6
Scott et al. *Foot Ankle Spec* [5]	IV, case series	ProToe^®^	63/NA	NA	NA	NA	NA	NA	0

*NA* not available, *AOFAS* American Orthopaedic Foot and Ankle Society Forefoot score

## Results

The results from the nine articles included in the work are reported in Table 2.

The satisfaction of the patient, taken into consideration in four publications, reports a good/excellent result in 93–100 % of the cases [11–13, 15].

In contrast, radiological arthrodesis is achieved in 60.5–100 % of the cases [4, 11]. This value result is heterogeneous and is barely correlated to the review cases, demonstrating the frequent establishment of a fibrous union.

Three publications compare the new devices to the K-wire: two of these use the traditional technique and one the buried technique. The Angirasa et al. and Roukis et al. publications report more satisfaction for the devices, yet none of these works cite any cases of revision [10, 11]. The Scholl et al. [17] group, instead, reports no significant difference of revisions utilizing K-wire with the buried technique (8.6 % against 10.7 % *p* = 0.754).

The AOFAS score (evaluated in three publications) shows a delta of 19, 45 and 58 points [12, 13, 15].

Minor complications, often asymptomatic and radiologically identified have been: malunion (2.4–7 %) [5, 10], displaced fixation (1.5–13 %) [10], mallet toe (2–23 %) [10, 13], non union (1.5 %) [16], hardware failures (3–5 %) [12, 16] and ruptures (5 %) [12].

The major complications, which required a surgical revision, vacillate between 0 and 8.6 %. These complications were mainly due to malunion, breaks or recurrence.

In conclusion, only two works took the price of the devices into consideration; Coillard et al. reported a 20 times higher price of these devices compared to the K-wire [13]. Ellington et al., instead, reported a price of $225 per device (StayFuse™, Nexa Orthopaedics, San Diego, CA) [14].

## Discussion

Although hammertoe is a very frequent disease, the treatment is still heavily disputed. In the structured deformities not suited for manual correction, PIP fusion is considered, today, the standard treatment [4]. The K-wire technique is the most utilized method for performing the fusion, as it is fast, cheap and simple to implant [3]. On the other hand, this kind of fixation method also has weak points: the exterior communication that predisposes for infections and traumatisms, the violation of the distal inter-phalangeal (DIP) joint, the lack of compression and rotational control and, finally, discomfort at removal [3, 7–10].

Because of this, the intramedullary devices aim to solve the weak points of the K-wire technique. Indeed, the results reported above seem to be slightly better than those of the K-wire, especially regarding patient satisfaction and malalignment of the arthrodesis.

Considering everything, the type of complications reported for the new devices and the K-wire treatment have been similar, save the superficial infections. Taking into consideration the major complications, in other words the cases which needed a reoperation in the articles that directly compare the new devices to the K-wire, no differences were found [10, 11], or in any case no statistically significant differences [17].

On the other hand, as reported by Ellington and Coillard, the devices definitely have higher prices compared to the K-wire, which represents a limit to their utilization, especially in the case of multiple toe corrections [13, 14].

Currently, no evidence exists in the literature which justifies the use of these new devices, especially considering their high price. For this reason cost-benefit studies are necessary to understand whether lower reoperation rates can justify the use of these devices as the new standard treatment in the future for hammertoes.

Regarding reoperation, this can also result in difficulties, especially in the phase of the removal of the device, and cause an excessive reduction of the toe length. For this reason new materials such as PEEK aim to make the revision easier.

Of the 16 devices currently available on the US and EU markets, as reported in Table 2, only four are also described in the literature (according to the criteria previously mentioned) and did not show significant differences in their results. For the remaining devices, future studies are still necessary.

In conclusion, the new intramedullary devices represent an interesting topic because of their continuous evolution and the constant birth of new devices on the market with new characteristics and material compositions.

The use of these devices seem to provide good results; however, the dilemma tied to their high price is not negligible. For this reason, cost-benefit studies that are still lacking in the literature seem necessary to justify the supremacy and the use of the new devices in the future as standard treatment for hammertoes.
